# Effects of a blood-free mosquito diet on fitness and gonotrophic cycle parameters of laboratory reared *Anopheles gambiae* sensu stricto

**DOI:** 10.1186/s13071-024-06345-y

**Published:** 2024-07-06

**Authors:** Faith Allan Mosi, Isaack Rutha, Rita Velez, Johnson Kyeba Swai, Yeromin P. Mlacha, Joana Marques, Henrique Silveira, Brian B. Tarimo

**Affiliations:** 1 Environmental Health and Ecological Science Department, Ifakara Health Institute-Bagamoyo Office, P.O. Box 74, Bagamoyo, Tanzania; 2 School of Life Sciences and Bioengineering, The Nelson Mandela African Institution of Science and Technology (NM-AIST), P.O. Box 447, Arusha, Tanzania; 3 Global Health and Tropical Medicine, GHTM, Associate Laboratory in Translation and Innovation Towards Global Health, LA-REAL, Instituto de Higiene e Medicina Tropical, Universidade Nova de Lisboa, IHMT-NOVA, Rua da Junqueira 100, 1349-008 Lisbon, Portugal; 4 Vector Biology Unit, Epidemiology and Public Health Department, Swiss Tropical and Public Health Institute, Kreuzstrasse 2, Allschwil, 4123 Basel, Switzerland; 5 University of Basel, Petersplatz 1, 4001 Basel, Switzerland

**Keywords:** Artificial diet, *Anopheles*, *Anopheles gambiae*, Mosquito fitness, Gonotrophic cycle, BLOODless

## Abstract

**Background:**

The current rise of new innovative tools for mosquito control, such as the release of transgenic mosquitoes carrying a dominant lethal gene and *Wolbachia*-based strategies, necessitates a massive production of mosquitoes in the insectary. However, currently laboratory rearing depends on vertebrate blood for egg production and maintenance. This practice raises ethical concerns, incurs logistical and cost limitations, and entails potential risk associated with pathogen transmission and blood storage. Consequently, an artificial blood-free diet emerges as a desirable alternative to address these challenges. This study aims to evaluate the effects of a previously formulated artificial blood-free diet (herein referred to as BLOODless) on *Anopheles gambiae* (*An*. *gambiae* s.s.; IFAKARA) gonotrophic parameters and fitness compared with bovine blood.

**Methods:**

The study was a laboratory-based comparative evaluation of the fitness, fecundity and fertility of *An*. *gambiae* s.s. (IFAKARA) reared on BLOODless versus vertebrate blood from founder generation (F_0_) to eighth generation (F_8_). A total of 1000 female mosquitoes were randomly selected from F_0_, of which 500 mosquitoes were fed with bovine blood (control group) and the other 500 mosquitoes were fed with BLOODless diet (experimental group). The feeding success, number of eggs per female, hatching rate and pupation rate were examined post-feeding. Longevity and wing length were determined as fitness parameters for adult male and female mosquitoes for both populations.

**Results:**

While blood-fed and BLOODless-fed mosquitoes showed similar feeding success, 92.3% [95% confidence interval (CI) 89.7–94.9] versus 93.6% (95% CI 90.6–96.6), respectively, significant differences emerged in their reproductive parameters. The mean number of eggs laid per female was significantly higher for blood-fed mosquitoes (*P* < 0.001) whereas BLOODless-fed mosquitoes had significantly lower hatching rates [odds ratio (OR) 0.17, 95% CI 0.14–0.22, *P* < 0.001]. Wing length and longevity were similar between both groups.

**Conclusions:**

This study demonstrates the potential of the BLOODless diet as a viable and ethical alternative to vertebrate blood feeding for rearing *An*. *gambiae* s.s. This breakthrough paves the way for more efficient and ethical studies aimed at combating malaria and other mosquito-borne diseases.

**Graphical Abstract:**

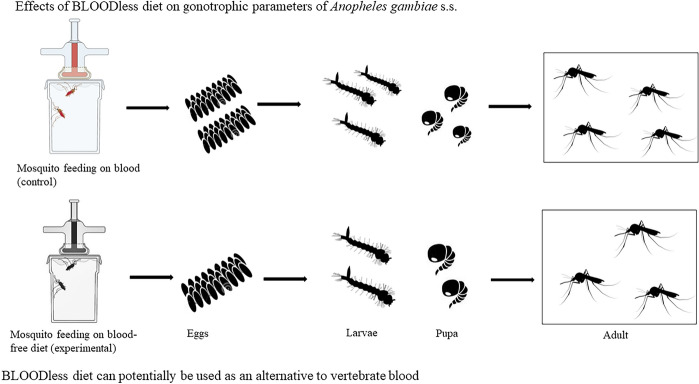

**Supplementary Information:**

The online version contains supplementary material available at 10.1186/s13071-024-06345-y.

## Background

Malaria control efforts, as well as the current rise of other mosquito-borne diseases (such as dengue, encephalitis, chikungunya, Zika and yellow fever), have resulted in the development of several innovative vector control tools for genetic biocontrol of vectors, such as genetically engineered mosquitoes [1, 2], which rely on the release of mosquitoes produced in captivity into the wild. For these novel techniques to be successful, the rearing program must be able to generate often large numbers of insects with normal behaviour and physical fitness.

Most disease transmitting mosquitoes are anautogenous females [3] meaning that they require a vertebrate bloodmeal for egg development and maturation. This characteristic makes it challenging to produce mosquitoes in large-scale owing to (i) ethical concerns (such as animal welfare regulations that emphasize the use of non-animal protocols), (ii) risk of pathogen transmission from human blood, or animal blood from a slaughterhouse that may be screened for a few known pathogens and (iii) budgetary constraints associated with the acquisition of vertebrate blood [4]. Several protocols on mosquito rearing and feeding methods based on vertebrate blood have been published [5, 6]. One major drawback of all the techniques is that they all need live animals and/or drawn blood. In certain countries, extensive work is required to get approval to use live animals [4]. Significant limitations also apply to donated human blood because (i) it bears the risk of blood-borne infections, (ii) it is subjected to stricter rules compared with animal blood [7], and (iii) it can be costly to obtain and difficult to preserve for long periods of time [4]. To develop a substitute for vertebrate blood for mosquito rearing and research, artificial diets that mimic a bloodmeal must be developed and shown to be efficient at producing fit progeny.

It has been demonstrated that artificial diets based on the rich nutrient content of human blood stimulate oogenesis and fertility in female mosquitoes in vivo, but with less efficacy than a conventional vertebrate bloodmeal [4, 8, 9]. Baughman et al. [10] successfully reared *Anopheles* mosquitoes on a plasma supplemented with adenosine triphosphate (ATP), but with lower reproductive capacity compared with blood-fed mosquitoes. Another approach utilises bovine serum albumin (BSA) as the primary protein source in artificial meals [11]. While this method is effective, BSA might be more expensive than whole blood, depending on the source and supply chain. Nonetheless, blood has a shelf life of 2–4 weeks, depending on the anticoagulant used, and it must be obtained on a regular basis, increasing the overall cost. Thus, the animal source of blood or anticoagulant used has an impact on mosquito fecundity and viability [12].

A recent study showed that the blood-free diet, BLOODless [13] tested in this study had comparable outcomes to blood-fed colonies in terms of fecundity and fitness, representing a significant improvement in artificial blood-free meals for rearing *Anopheles*. Thus, it was effective on *An*. *stephensi*, *An*. *aquasalis*, and *An*. *coluzzii* (former *An*. *gambiae* M form) Yaoundé strain [10]. However, evidence of its success in the rearing of *An*. *gambiae* s.s. for consecutive generations is yet to be demonstrated. This study examined the impact of BLOODless on the gonotrophic cycle and mosquito fitness parameters of a pyrethroid susceptible *An*. *gambiae* s.s. (IFAKARA) strain in Tanzania.

## Methods

### Mosquito rearing

*An*. *gambiae* s.s. (IFAKARA) were reared in an established insectary located at Ifakara Health Institute in Bagamoyo, Tanzania. Colonies were maintained, and all experiments were carried out at a constant temperature of 27 ± 2 °C and 80 ± 10% relative humidity. After each feeding, eggs were flushed into larvae rearing bowls using 200 mL of distilled water. After eggs hatched, fish food (Tetramin® Tropical Flakes) was added to the bowls for the successive 2 weeks until pupation of all larvae. Pupae were transferred from the rearing bowls into an emergence bowl with a plastic Pasteur pipette and then placed in a netted cage for adult emergence. On the following day, the emergence bowl was examined for dead pupae, drowned adult mosquitoes, and empty puparia, all of which were discarded. Adult mosquitoes were provided with 10% sucrose ad libitum in a holder container with filter paper. The containers were then removed from the cage 12 h prior to being offered a blood or BLOODless feeding.

### Origin of blood and BLOODless diet source

Bovine blood was obtained from the cows raised at Ifakara Health Institute. The lyophilized BLOODless (blood-free diet) was donated by Global Health and Tropical Medicine/Instituto de Higiene e Medicina Tropical-Universidade Nova de Lisboa (GHTM/IHMT-NOVA), Portugal. The diet is composed of a protein source, glucose, cholesterol, a phagostimulant, amino acids, and vitamins. Detailed diet description can be found on Marques et al. [13].

### Membrane-feeding technique

A water-jacketed system was used for feeding. The water bath was set to 38 °C and connected to glass feeders via tubing. In the glass feeder wells, 0.2 mL of bovine blood or BLOODless was added. Rubber bands were used to hold Parafilm™ membranes in place after they were stretched across the feeder bottom (Fig. S1 1a). Cups of mosquitoes were placed underneath the feeders, making sure that the bottom of each feeder touched the netting on top of the cup (Fig. S1 1b &1c). After 30 min of feeding, the number of mosquitoes that were fully engorged was recorded and mosquitoes that did not feed or partially fed were discarded. Mosquitoes were provided with 10% sucrose ad libitum 24 h after membrane feeding.

### Effect of BLOODless on *An*. *gambiae* s.s. (IFAKARA) gonotrophic cycle parameters

From the founder generation (F_0_) of laboratory reared *An*. *gambiae* s.s. (IFAKARA), 1000 5 day old female mosquitoes were selected for the study. Half of them were fed bovine blood (hereinafter blood-fed colony) and the remaining 500 females were fed with BLOODless (hereinafter BLOODless-fed colony) for egg production. The feeding was performed once on 5 day old female mosquitoes. The feeding experiments were consistently conducted in a dark room at 1 p.m. Unfed mosquitoes were removed, and fully engorged mosquitoes were kept in the cage at 27 ± 1 °C, 75% relative humidity. An egg-plate for oviposition was introduced into the rearing cages 48 h post-feeding. After 48 h, the eggs that had been laid were counted and flushed into a bowl with water for incubation and subsequent hatching. The resulting larvae were followed until pupae which then emerged into adults of the next generation. These steps were repeated from F_0_ to F_8_ generation.

During the rearing process, the following parameters were evaluated for both the blood- and BLOODless-fed colonies:

1. Feeding rate: immediately post-feeding, all fully engorged females were counted, the mosquitoes that failed to engorge or partially fed were counted and discarded and calculated as the number of fully engorged females/total number of females.
2. Survival rates of engorged females at 48 h post-feeding were recorded before placing the oviposition plates.
3. Fecundity was measured by: (i) observing egg development stages 72 h post-feeding by dissecting 20 randomly selected females from those that successfully fed. The development was classified following the Christopher’s stages of egg development in *Anopheles* [14]; and (ii) calculating the number of laid eggs per female by counting the eggs laid on the egg-plates 72 h post-feeding.
4. Fertility was evaluated by observing hatchability of the eggs laid 48 h post-oviposition by counting the number of L_1_ compared with the number of laid eggs.

### Effect of BLOODless on *An*. *gambiae* s.s. (IFAKARA) adult fitness

#### Longevity

A total of 20 male and female adult mosquitoes from each generation were collected and transferred to a specific cage on the day of emergence (registered as the eclosion date). Each day adults’ deaths were registered, and longevity was calculated after the last mosquito died, for both the BLOODless-fed and blood-fed colonies. These adult mosquitoes were maintained under standard insectary conditions with access to 10% sucrose ad libitum.

#### Wing length

The procedure for wing length was adapted from Marques et al. [15]. The 5-day old male and female mosquitoes (*n* = 20) from each generation were collected and anaesthetized at −20 °C for at least 90 s. Under a stereoscope, the thorax of each mosquito was gently grasped with forceps and placed ventral side up. Both wings were cut at the point where they attach to the thorax using a scalpel. They were then collected and placed on a clean microscope slide with a dried drop of Entellan mounting medium. Using a 20G needle, extra mounting media was added to the borders of a coverslip which was then placed on the wings. The wing length was measured from the distal end of the alula to the wing tip excluding fringe scale under a stereoscope, using a micrometer.

### Statistical analysis

Gonotrophic cycle and mosquito fitness parameters were evaluated for both blood-fed and BLOODless-fed colonies as follows: the feeding rate was determined by calculating the proportion of fed mosquitoes compared with the total number of females in the cage; the fecundity was evaluated through two different methods: (i) the mean number of mature eggs (stage V) present on dissected mosquitoes, and (ii) the mean number of laid eggs per female mosquito was determined by dividing the total number of eggs observed by the total number of females; the fertility was evaluated by counting the total number of *L*_1_ 48 h after egg incubation and dividing it by the total number of eggs laid; finally, the pupation rate was determined by comparing the total number of pupae and the initial number of *L*_1_.

Adult longevity was quantified by the average number of days that the randomly selected 20 mosquitoes lived. In addition, the wing length was measured from the 20 randomly selected male and female mosquitoes and presented separately as an average by sex.

Statistical analysis was performed using STATA 17 software (StataCorp LLC, USA). Descriptive statistics mean or proportions with 95% confidence intervals (95% CI) were calculated. Regression analysis with meal, sex and generation added as categorical fixed factors was carried out to determine differences on the estimated gonotrophic cycle and fitness parameters between the *An*. *gambiae* s.s. colonies fed either with BLOODless or bovine blood. Odds ratios (OR) for proportional outcomes (feeding rate, mortality and hatching rates) and incidence rate ratios (IRR) for continuous outcomes (fecundity, longevity, wing length) were estimated from respective logistic and linear regression models. Likelihood ratio test showed that the models including both sex and generation as fixed factors were better than those having either sex or generation alone (*P* < 0.001). The presented results are combined for eight generations.

For logistic reasons, we chose to perform only one replicate of the experiment (all eight generations), which reflects on independent replicates per generation, and might have some impact on the statistical significance of the results.

## Results

### Feeding rate

A total of 3600 *An*. *gambiae* s.s. mosquitoes were offered bovine blood, while 3586 were offered BLOODless. The mean percentage of the engorged female mosquitoes fed on BLOODless was 93.6% (95% CI 90.6–96.6; *n* = 3326), while those fed on blood was 92.3% engorgement (95% CI 89.7–94.9; *n* = 3312). The results of the logistic regression that adjusted for mosquito generation indicated that the feeding rates of BLOODless and bovine blood did not differ significantly (*P* = 0.488; Table 1).

**Table 1:** Summary of gonotrophic parameters of *Anopheles gambiae* s.s. females fed on blood or BLOODless diet

Outcome	Meal	Proportion^a^/mean^b^ (95% CI)	OR^a^/IRR^b^ (95% CI)	*P*-value
Feeding success	Blood	92 (90–95)^a^	1	0.488
	BLOODless	94 (91–97)^a^	1.12 (0.82–1.53)^a^	
Post-feeding Mortality	Blood	12 (7–17)^a^	1	0.636
	BLOODless	15 (7–23)^a^	1.12 (0.70–1.79)^a^	
Stage V eggs per female	Blood	56 (52–60)^b^	1	< 0.001*
	BLOODless	46 (42–50)^b^	0.00 (0.00–0.01)^b^	
Eggs laid per female	Blood	36 (27–45)^b^	1	< 0.001*
	BLOODless	26 (17–33)^b^	0.00 (0.00–0.01)^b^	
Hatch Rate	Blood	79 (73–85)^a^	1	< 0.001*
	BLOODless	37 (33–42)^a^	0.17 (0.14–0.22)^a^	
Pupation Rate	Blood	90 (87–98)^a^	1	0.351
	BLOODless	93 (88–98)^a^	1.45 (0.66–3.17)^a^	

*OR* odds ratio estimated from logistic regression, *IRR* incidence Rate ratios estimated from linear regression

^a^Represents proportions in percentage

^b^Represents arithmetic mean

### Post-feeding mortality

Before oviposition, 11.9% (95% CI 6.9–16.8) of mosquitoes that had previously fed on bovine blood died (420/3312), compared with 14.7% (CI 6.6–22.8) of mosquitoes that fed on BLOODless (456/3326). Controlled for mosquito generation, logistic regression showed no indication of a significant difference in post-feeding mortality between mosquitoes fed on both meals (Table 1).

### Fecundity

A total of 174 and 178 mosquitoes that were fully engorged on bovine blood or BLOODless, respectively, were dissected. A total of 9761 and 8195 stage V eggs were observed from blood-fed and BLOODless-fed female mosquitoes, respectively. The mean number of mature eggs per female from the blood-fed colony was 56 eggs (95% CI 52–60) and that of the BLOODless-fed colony was 46 eggs (95% CI 42–50). Linear regression adjusting for mosquito generation showed that there is significantly higher number of fully developed eggs after 48 h in blood-fed compared with BLOODless-fed mosquitoes (Table 1). The mean number of eggs laid per female was 36 eggs (95% CI 27–45) for mosquitoes fed on bovine blood while for those fed on the BLOODless was 26 eggs (95% CI 17–33). The results of a linear regression controlling for mosquito generation indicated a significantly higher number of eggs laid per female in mosquitoes fed on blood compared with BLOODless (Table 1).

### Fertility

A total of 71,836 and 24,056 *L*_1_ larvae hatched from eggs of mosquitoes fed on bovine blood and on BLOODless, respectively. The mean hatching rate of eggs from mosquitoes fed on bovine blood was 79.2% (95% CI 73.7–84.6), while those from BLOODless-fed mosquitoes was 37.4% (95% CI 33.3–41.5; Table 1). The results of a logistic regression, with mosquito generation kept constant, indicated evidence of a difference in egg hatching rates. Mosquitoes fed on BLOODless had an 83% lower hatching rate compared with those fed on bovine blood (Table 1).

The rates of development of larvae to pupae from mosquitoes fed on bovine blood was 90.3% (95% CI 87.1–93.5) compared with 92.6% (95% CI 87.6–97.7) from BLOODless-fed mosquitoes. Logistic regression controlling for mosquito generation showed no evidence of a difference in larvae conversion rates between mosquitoes fed on BLOODless and those fed on bovine blood (Table 1).

### Adult fitness

#### Longevity

The average life expectancy for adults from the blood-fed colony was 20.7 days (95% CI 19.6–21.8) for males and 18.8 days (95% CI 17.8–19.9) for females. Whilst male adults from the BLOODless-fed colony lived for 20.7 days (95% CI 19.5–22.0) and the females lived on average 18.3 days (95% CI 17.1–19.4; Table 2). The results of a linear regression, with mosquito sex and generation controlled for, showed no statistically significant difference on life span between mosquitoes fed on BLOODless and those fed on bovine blood (Table 2). After controlling for meal and generation, there was evidence that males generally lived longer than females (Table 2).

**Table 2:** Summary of fitness parameters of *Anopheles gambiae* s.s. raised on blood or BLOODless

Outcome	Sex	Meal	Mean (95% CI)	IRR (95% CI)	*P*-value
Longevity	Female	Blood	18.8 (17.8–19.9)	0.77 (0.25–2.39)	0.656
		BLOODless	18.3 (17.1–19.4)		
	Male	Blood	20.7 (19.6–21.8)		
		BLOODless	20.7 (19.5–22.0)		
Wing length	Female	Blood	3.30 (3.24–3.37)	1.02 (0.99–1.06)	0.220
		BLOODless	3.40 (3.32–3.48)		
	Male	Blood	3.17 (3.11–3.23)		
		BLOODless	3.26 (3.19–3.32)		

#### Wing length

The mean wing length of female mosquitoes from the blood-fed colony was 3.30 mm (95% CI 3.24–3.37) while those from the BLOODless-fed colony averaged 3.40 mm (95% CI 3.32–3.48). The mean wing length of male mosquitoes in the blood-fed colony was 3.17 mm (95% CI 3.11–3.23) and that from the BLOODless-fed colony was 3.26 mm (95% CI 3.19–3.32) (Table 2). The results of a linear regression, with mosquito sex and generation kept constant, showed that there was no evidence indicating that the wing length of mosquitoes fed on the BLOODless were larger than those fed on bovine blood (Table 2). After controlling for mosquito meal and generation, there was evidence that on average females had larger wings than males (Table 2).

## Discussion

This study aimed to compare the effectiveness of a previously formulated blood-free diet (BLOODless) and bovine blood on rearing *Anopheles* *gambiae* s.s. in a laboratory in Tanzania. We hypothesized that there would be no evidence of a significant difference on the impact of feeding rates, fecundity (number of stage V eggs in ovaries or eggs laid per female), fertility (egg hatching rates), larvae pupation rates, longevity and wing length between mosquitoes reared on the BLOODless diet and those fed on bovine blood. Our results showed that the mosquitoes exclusively fed on BLOODless presented similar behaviour in terms of pupation rates, longevity and wing length compared with mosquitoes fed on bovine blood, while the hatching rate and the number of laid eggs per female were higher in the blood-fed colony.

We observed that female mosquitoes fed on BLOODless diet showed feeding rates that were not significantly different to those fed on blood; Marques et al., reported higher engorgement rates in various *Anopheles* species fed on this blood-free diet, suggesting that the BLOODless diet may be more attractive to female mosquitoes than mice blood [13, 15]. A possible reason is that mosquitoes frequently rely on sugar as their initial and primary energy source for flight and, the diet consists of a sugary solution enriched with amino acids and phagostimulant, that may attract the mosquitoes more towards the diet [8, 16]. Feeding choice experiments to determine whether mosquitoes will prefer the diet over vertebrate blood are required.

The survival of adult female mosquitoes 48 h post-feeding was similar for both the blood-fed and BLOODless-fed mosquitoes, showing that there was no evidence that the BLOODless causes preliminary death of mosquitoes, in accordance to what was previously observed on *An*. *coluzzii* [13, 15].

In terms of fecundity (number of laid eggs per female), we observed that blood-fed *An*. *gambiae* s.s laid more eggs than the BLOODless-fed females. Similar findings were observed when a different artificial diet was used in *An*. *darlingi* [17]. However, a previous study performed on *An*. *coluzzii* using the same BLOODless diet found that there was no significant difference between the number of eggs produced by the diet-fed females and the blood-fed mosquitoes [13, 15]. The differences could be owing to different larvae-diet composition, settings, species and study methodology used. In our study, the number of eggs were recorded in each generation from F_1_ to F_8_ and then analysed, while on previous publications the data were generated from three independent experiments. In addition, in previous studies [13, 15] BLOODless was prepared freshly before each feeding experiment, in the present study BLOODless was stored on its lyophilized form until use, which might impact the final results.

Fertility was affected by the BLOODless diet since fewer eggs produced by mosquitoes hatched. It has been reported that eggs from mosquitoes fed with BSA-based diets, such as the one used in this study, had a lower hatching rate compared with vertebrate blood. This fact suggests that supplementing the diet with additional nutrients to facilitate embryo development and survival of the eggs could be beneficial [4, 10, 11, 17, 18]. There is evidence that adding commercially available hemoglobin or iron (III) chloride as iron sources, respectively, to artificial diets would increase eggs viability and hatching rates [4, 19, 20]. Although these studies were performed on *Aedes aegypti*, the mechanism for oogenesis is similar in all female mosquitoes, thus adding such supplements to the current BLOODless diet might improve egg viability and hatching rates of *An*. *gambiae* s.s.

Our results showed no difference in pupation rates of larvae from the BLOODless-fed mosquitoes even though there is a higher concentration of isoleucine in the diet that is hypothesized to improve larvae pupation [21]. The findings differ from those of *An*. *coluzzi*, where larvae from BLOODless-fed mosquitoes had lower larval mortality compared with mice blood [13, 15].

The life expectancy and wing length of adult BLOODless-fed mosquitoes were similar to the bovine blood-fed mosquitoes for both male and female mosquitoes. However, we found that males lived longer than females for both colonies. These results differ from a previous study that tested the same diet and that showed that females lived longer than males [15]. The reason why males had a longer life span in our study is yet unknown.

According to Gonzales et al. [11], for an artificial diet to successfully replace vertebrate blood, it should meet the following criteria: (i) it must be able to attract females to fully engorge in substantial quantities, (ii) it should facilitate vitellogenesis (the process of egg development), (iii) it should allow for the production of large batches of eggs, (iv) the offspring competitiveness (such as blood seeking behaviour, mating, flight capacity) should be similar to that of wild mosquitoes, and finally, and more importantly, v) the meal should not adversely affect mosquito behaviour and immunity.

Findings from our study indicate that BLOODless can promote the healthy and successful development of viable eggs in female *An*. *gambiae* s.s., their hatching and growth from larvae to pupae and into adults, which is essential for sustaining mosquitoes in the laboratory. It has been observed that different vertebrate sources of a bloodmeal impact mosquito gonotrophic parameters and fitness [10, 12, 20]. Artificial diets like the one tested in this study offer the opportunity of creating balanced and stable meals for female mosquitoes containing all the essential nutrients for the development and maturation of eggs, as well as the aquatic stages to emergence onto adults. Furthermore, BLOODless serves as an ethical substitute for the utilization of vertebrate blood [20].

The BLOODless diet offers several advantages. First, it boosts chemical stability in comparison with vertebrate blood, which contains anticoagulants to prolong shelf life but may affect fecundity and fertility [4, 11]. Secondly, its usage aligns with animal welfare regulations by promoting the 3 R’s (replacement, reduction, and refinement), thus encouraging the utilization of non-animal protocols to minimize laboratory animal use. Despite initial higher costs for BLOODless ingredients, it can ultimately reduce operational expenses associated with animal husbandry. The cost per millilitre depends on the amount of bovine serum albumin (BSA) purchased and ranges from US$0.17 to US$0.34 [4]. On the other hand, whole blood’s pricing varies on the basis of animal source and blood treatment method. For instance, defibrinated blood from Haemostat Laboratories may cost: bovine, UD$0.14–0.60, sheep, US$0.05–0.38, and rabbit, US$0.39–0.73 [4]. Consequently, the unit price of whole blood is comparable to that of a BSA-based artificial diet.

We recommend further studies to validate the effectiveness of BLOODless for rearing *Anopheles funestus* – a competent malaria vector that is highly resistant to pyrethroids and other public insecticides – and *Anopheles arabiensis* – a secondary vector that is also highly resistant to public health insecticides – among other laboratory strains and populations collected from the field. Other tests, such as host-seeking behaviour in females as well as mating success, flight capacity and mating competitiveness of males, should also be conducted before implementation in insectaries worldwide.

## Conclusions

Our study adds to the body of evidence that blood-free diets have the potential to substitute vertebrate blood in rearing mosquitoes in laboratories. BLOODless proved to be effective in attracting female mosquitoes, supporting crucial reproductive processes, producing fit egg batches and showed normal mosquito biology. However, mosquitoes reared exclusively on BLOODless showed lower hatch rates and fecundity. Despite some of the promising results observed, further experimentation with larger samples sizes, various mosquito species and prolonged time frame are required on BLOODless before it can be definitively applied as an alternative to blood for rearing mosquitoes.

### Supplementary Information

**Additional file 1: Dataset S1.** Gonotrophic parameters of *An*. *gambiae* s.s*.* mosquitoes fed on either blood or BLOODless.

**Additional file 2**: **Figure S1.** Mosquito feeding procedures. (**a**) Feeding preparation: the water bath was set to 38 ºC and connected to glass feeders via tubing, the cups were place underneath the feeders to allow access to blood or BLOODless; (**b**) Glass feeder containing the BLOODless and mosquito feeding through parafilm membrane; and (**c**) Glass feeder containing the bovine blood and mosquito feeding through parafilm membrane

## Data Availability

The data generated in this study are available as Additional file 1.
