# Supplementary figures and images for: Effects of a blood-free mosquito diet on fitness and gonotrophic cycle parameters of laboratory reared Anopheles gambiae sensu stricto
Source: Parasit Vectors. 2024 Jul 6;17:289. doi: 10.1186/s13071-024-06345-y (PMC11227146; doi:10.1186/s13071-024-06345-y)

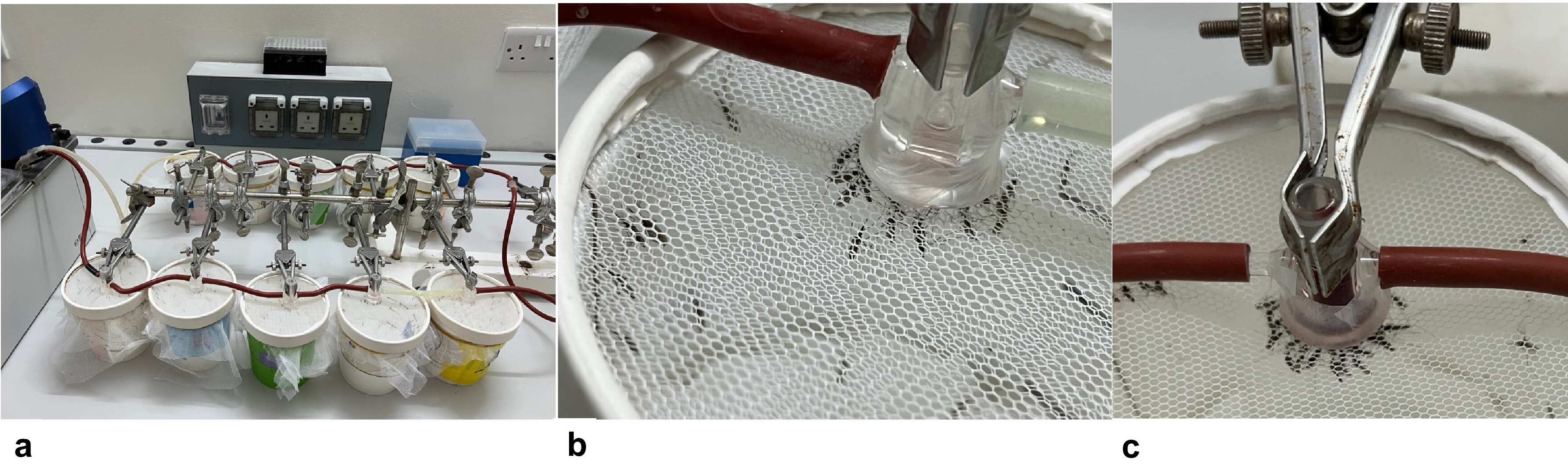

Supplement: Supplementary file 2 — Additional file 2: Figure S1. Mosquito feeding procedures. (a) Feeding preparation: the water bath was set to 38 ºC and connected to glass feeders via tubing, the cups were place underneath the feeders to allow access to blood or BLOODless; (b) Glass feeder containing the BLOODless and mosquito feeding through parafilm membrane; and (c) Glass feeder containing the bovine blood and mosquito feeding through parafilm membrane [file 13071_2024_6345_MOESM2_ESM.jpg]
